# The sinemydid turtle *Ordosemys* from the Lower Cretaceous Mengyin Formation of Shandong, China and its implication for the age of the Luohandong Formation of the Ordos Basin

**DOI:** 10.7717/peerj.6229

**Published:** 2019-01-15

**Authors:** Da-Qing Li, Chang-Fu Zhou, Lan Li, Jing-Tao Yang, Longfeng Li, Márton Rabi

**Affiliations:** 1 Institute of Vertebrate Paleontology and College of Life Science and Technology, Gansu Agricultural University, Lanzhou, Gansu, China; 2 School of Earth Sciences and Resources, China University of Geoscience (Beijing), Beijing, China; 3 College of Earth Science and Engineering, Shandong University of Science and Technology, Qingdao, Shandong, China; 4 School of Earth and Space Sciences, Peking University, Beijing, China; 5 Central Natural Science Collections, Martin-Luther Universität Halle-Wittenberg, Domplatz, Germany; 6 Department of Geosciences, University of Tübingen, Tübingen, Germany

**Keywords:** Early Cretaceous, Mengyin Formation, Sinemydidae, *Ordosemys*, Luohandong Formation

## Abstract

Chronostratigraphic correlation of terrestrial Early Cretaceous biotas in China is highly problematic due to the lack of marine deposits, few absolute dates, and limited number of index fossils. This often leaves vertebrate faunas as one of the few potential tools for a preliminary biostratigraphy. Taxonomic identity of fragmentary fossils is, however, often uncertain and many faunas are insufficiently sampled. Turtles are one of the most common elements of Early Cretaceous biotas of Asia and their skeleton is frequently preserved more completely than that of other vertebrates- they yet receive little attention from vertebrate paleontologists. We here record the presence of the sinemydid turtle *Ordosemys leios* from the Lower Cretaceous Mengyin Formation of Shandong Province, China, best known for the first dinosaurs and Mesozoic turtles described from the country. *Ordosemys* is the third turtle reported from the Mengyin Formation along with *Sinemys lens* and *Sinochelys applanata* and the only other formation where *Ordosemys* is known to co-occur with *Sinemys* is the Luohandong Formation of the Ordos Basin (Inner Mongolia), the type and so far only horizon of *Ordosemys leios*. The presence of the crocodyliform *Shantungosuchus* may further define a fauna that is so far only known from these two formations. The stratigraphic position of the Luohandong Formation is poorly controlled and it has been placed anywhere between the Valanginian and Aptian. Published absolute dates from the Mengyin Formation and the numerous shared vertebrate and invertebrate taxa (now also including turtles) implies a Valanginian—early Hauterivian age for the Luohandong Formation—in contrast to late Hauterivian-Albian as previously proposed using the temporal distribution of *Psittacosaurus*. The new specimen of *Ordosemys leios* preserves the only known manus of this species and ecomorphological analysis of limb proportions implies that it was a less capable swimmer compared to *Ordosemys liaoxiensis* coming from the younger Jehol Biota.

## Introduction

The Lower Cretaceous Mengyin Formation of Ningjiagou, Xintai, Shandong Province, China yielded one of the first non-avian dinosaurs described from China during the Sino-Swedish expeditions of 1916–1927 (the sauropod *Euhelopus zdanskyi* (Wiman, 1929)). Other vertebrates reported include amiiform and osteoglossomorph fishes, turtles, a stegosaurian, a theropod, and pterosaurs (e.g., Wiman, 1930; Dong, 1992; Wilson & Upchurch, 2009; Poropat & Kear, 2013; Borinder, Poropat & Kear, 2016). The age of this formation has been contested and it was initially regarded Upper Jurassic and then subsequently interpreted as Barremian or Aptian (e.g., Dong, 1992; Wu, Brinkman & Lu, 1994; Barrett & Wang, 2007; Wilson & Upchurch, 2009). There is growing consensus that the Mengyin Formation is Lower Cretaceous and recent zircon dating has resulted in an age of 145–136 Ma which is equivalent to the Berriasian-Valanginian interval of the basal Cretaceous (Xu & Li, 2015). The Mengyin Formation has yielded two turtles, the iconic bizarre species with a pair of lateral spines on the carapace, *Sinemys lens* Wiman, 1930 and the enigmatic *Sinochelys applanata* Wiman, 1930 (Hirayama, Brinkman & Danilov, 2000; Sukhanov, 2000). *Scutemys tecta* Wiman, 1930 from the same locality has been synonymized with *Sinochelys applanata* (Chkhikvadze, 1983, see also Sukhanov, 2000). The name *Sinemys lens* was subsequently used to conceptualize a larger group of Asian pan-cryptodiran Mesozoic turtles, Sinemydidae Yeh, 1963 later defined as a clade by Rabi et al. (2014).

**Figure 1:**
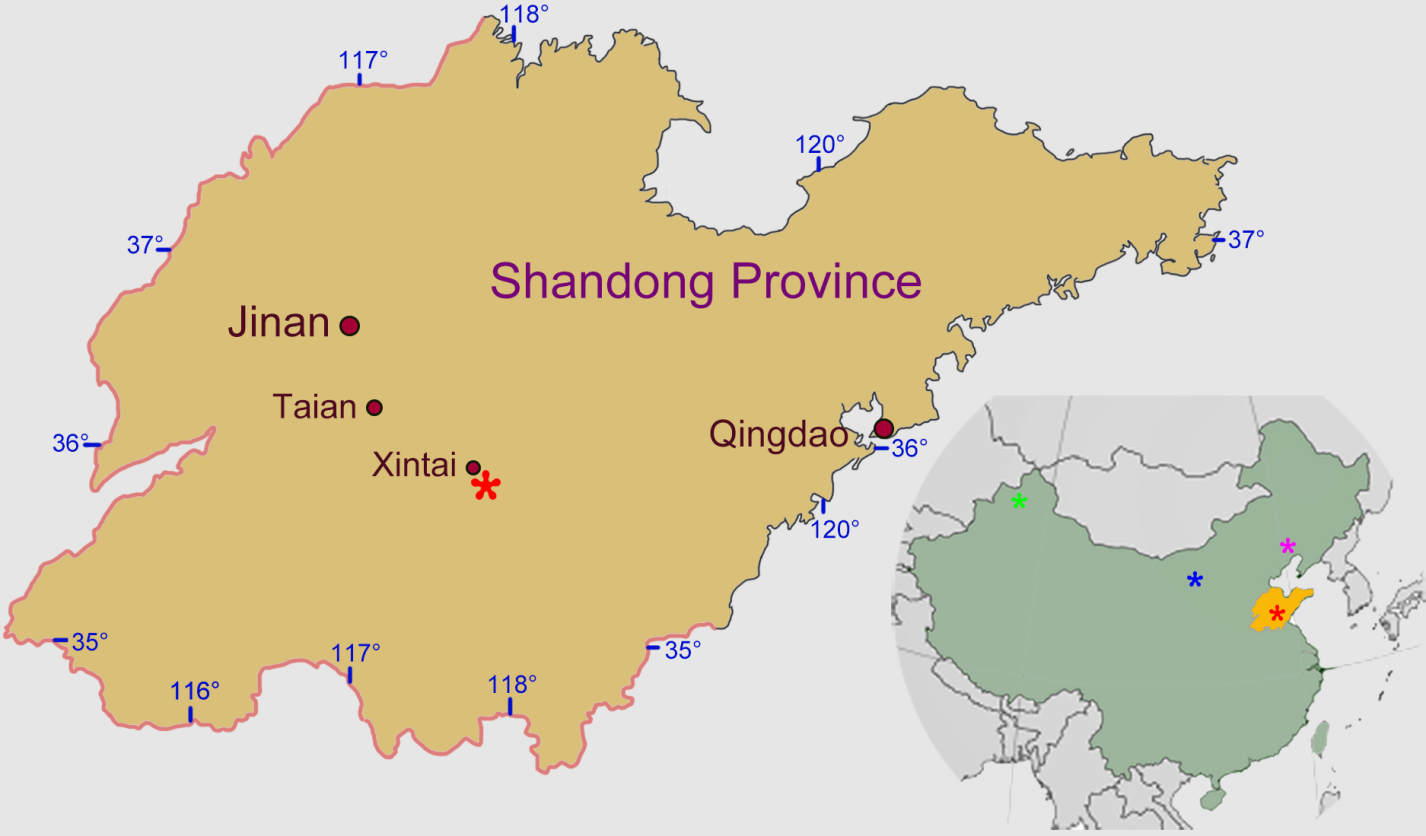
Fossil localities of *Ordosemys* spp. in the Early Cretaceous North China. The locality of *Ordosemys leios* (Red asterisk; 35°49′13″N; 117°49′30″E ) in Ningjiagou village, Xintai City, western Shandong Province; other localities of *O*. *liaoxiensis* (purple asterisk) from Yixian Formation of western Liaoning, *O*. *leios* (blue asterisk) from the Luohandong Formation of Inner Mongolia, and *O*. *brinkmania* (green asterisk) from the Lianmuqin Formation of eastern Xinjiang. Area map was modified from Zhou et al. (2017).

Following a lengthy hiatus in collecting from the Mengyin Formation, a slab containing five partial to near-complete turtle skeletons belonging to two taxa as well as remains of the fishes *Sinamia* and *Lycoptera* was recently found ca. 500 m North of Ningjiagou village. One of the turtle skeletons belong to *Sinemys lens* and the rest show great similarity with *Ordosemys leios* from the Luohandong Formation of Inner Mongolia. The description of these *Ordosemys* remains provided below represents the first study of new vertebrate material from the Mengyin Formation since the Swedish and early Chinese (Young, 1935) expeditions. Among freshwater sinemydid turtles, *Ordosemys* spp. is considered to have the broadest distribution ranging from eastern Xinjiang to western Liaoning in North China (Fig. 1). Besides the type species *Ordosemys leios* (Brinkman & Peng, 1993a; see also Brinkman & Wu, 1999), *O*. *liaoxiensis* was described from the lacustrine deposits of Yixian Formation of Beipiao, western Liaoning (Ji, 1995; Tong, Ji & Ji, 2004) and *O*. *brinkmania* from the fluvial deposits of the Tugulu Group of Wuerho, eastern Xinjiang (Danilov & Parham, 2007). In this work, we explore the implication of the new *Ordosemys* remains from the Mengyin Formation to the age of the Luohandong Formation of the Ordos Basin (Inner Mongolia). Furthermore, the preserved manus of the specimen provides an opportunity to evaluate the ecology of *Ordosemys leios* using published osteological correlates (Joyce & Gauthier, 2004).

## Material and Methods

**Figure 2:**
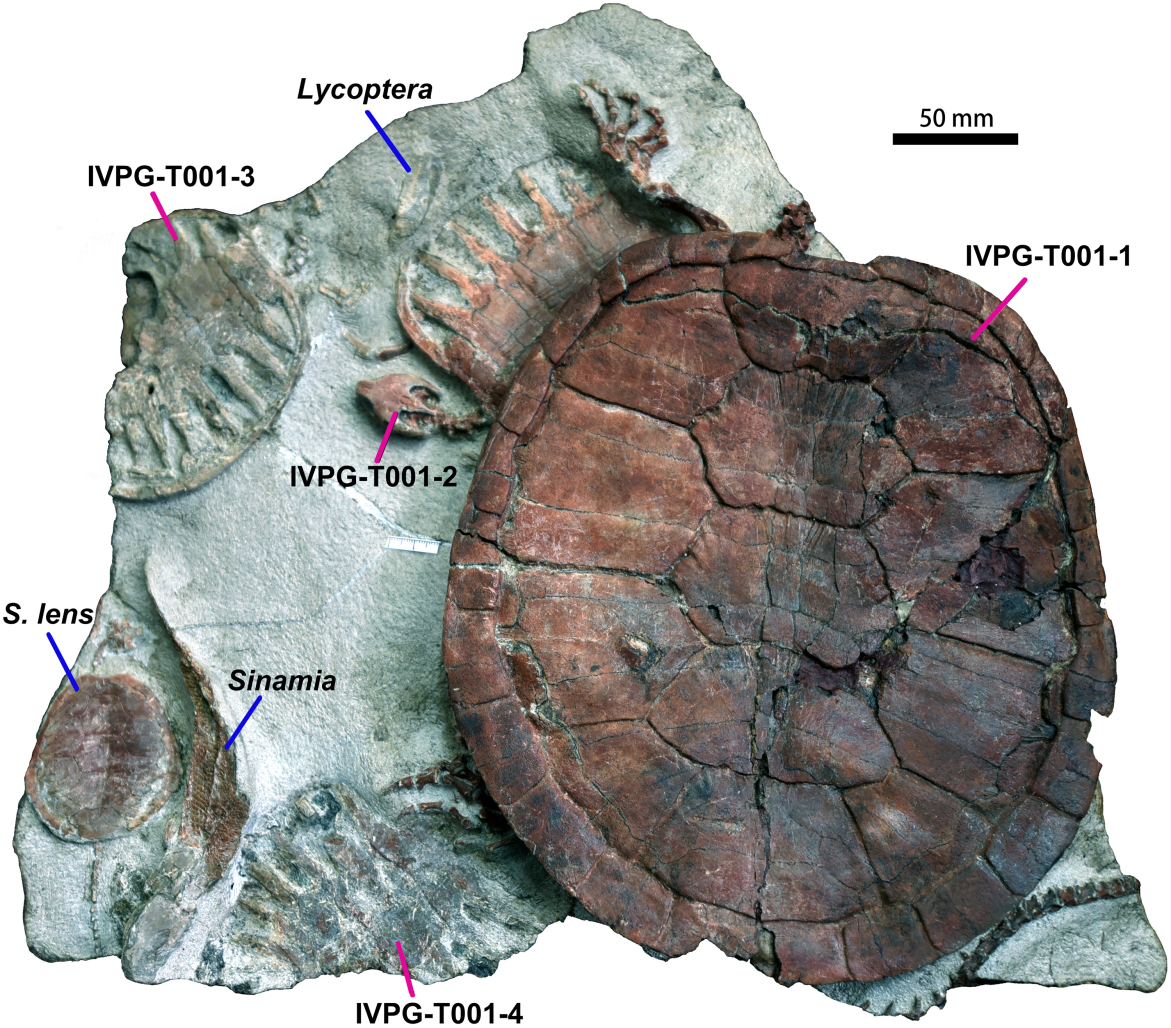
*Ordosemys leios* (IVPG-T001-1, IVPG-T001-2, IVPG-T001-3, and IVPG-T001-4; in dorsal view) from the Early Cretaceous Mengyin Formation of Ningjiagou, Xintai, western Shandong, China. Associated vertebrates in the fossil block (IVPG-T001) include the fishes *Lycoptera* and *Sinamia* and the other sinemydid turtle *Sinemys lens* (IVPG-T001-5).

**Figure 3:**
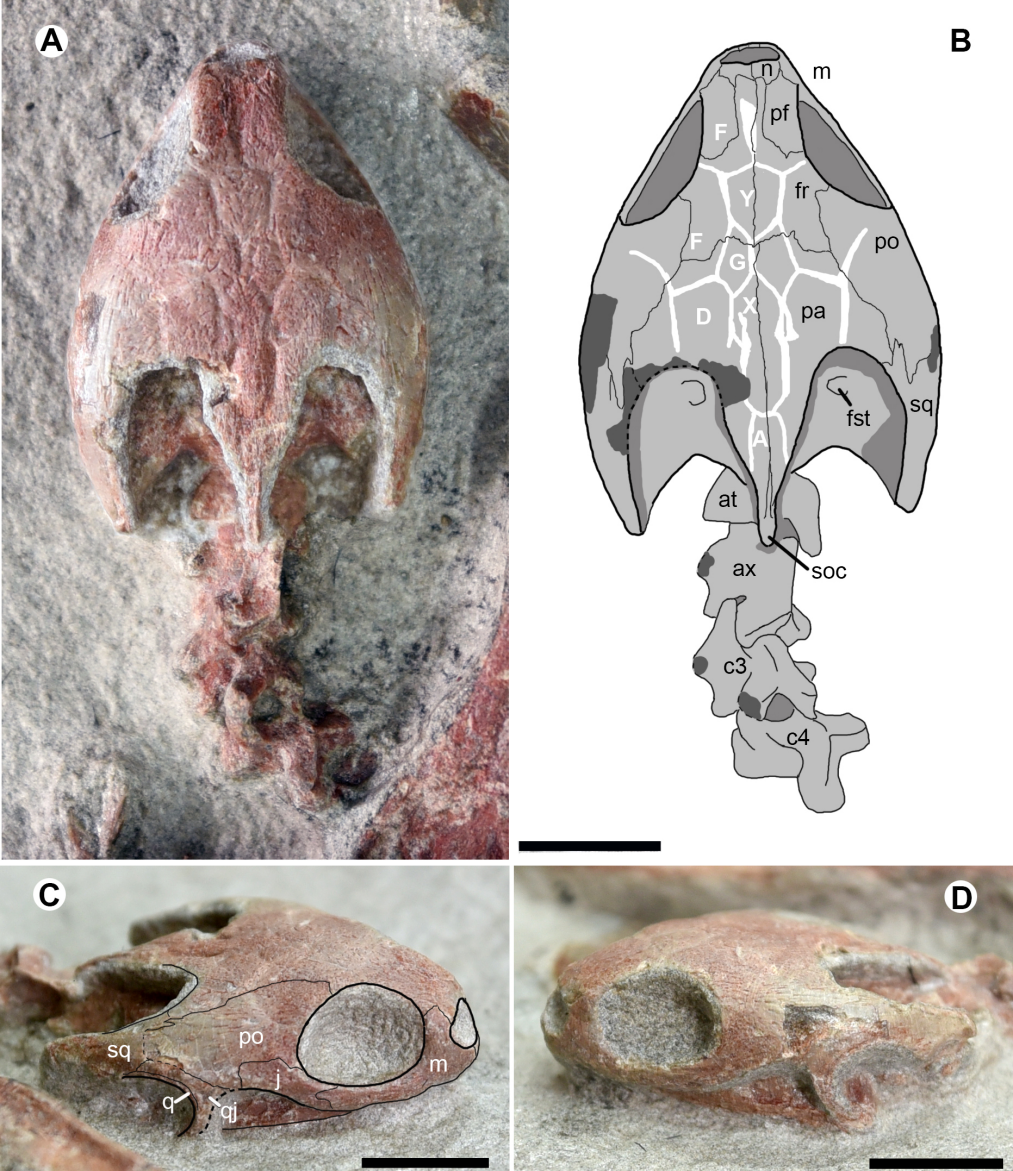
Cranial structure and cervical series of * Ordosemys leios* (IVPG-T001-2) from the Early Cretaceous Mengyin Formation of Ningjiagou, Xintai, western Shandong, China in dorsal (A and B) and lateral (C and D) views. Abbreviations: at, atlas; ax, axis; c3-c4, the third and fourth cervicals; fr, frontal; fst, foramen stapedio-temporale; j, jugal; m, maxilla; n, nasal; pa, parietal; pf, prefrontal; po, postorbital; q, quadrate; qj, quadratojugal; soc, supraoccipital crest; sq, squamosal; A, D, F, G, X, Y, scales of cranial roof. Scale bars equal to 10 mm.

Four *Ordosemys* specimens were recovered from the Lower Cretaceous Mengyin Formation at Ningjiagou, Xintai City, Shandong Province, China (Fig. 1). They were found in a single block (IVPG-T001), associated with a juvenile skeleton of *Sinemys lens* as well as three specimens of the fishes *Lycoptera* and *Sinamia* (Fig. 2). The fossils were prepared in the Institute of Vertebrate Paleontology of Gansu Agricultural University. Of these, the largest skeleton (IVPG-T001-1) is missing the cranium as well as some cervical and caudal vertebrae and represents an adult individual. The other three (IVPG-T001-2, T001-3 and T001-4) are juveniles in having smaller size and large costal-peripheral fenestrae (Figs. 2–3). A well-preserved skull is exposed in IVPG-T001-2 and the skull of IVPG-T001-4 is partially exposed under the shell of IVPG-T001-1. The juveniles are comparable in shell morphology and identified as the same taxon as the adult IVPG-T001-1 by the shell profile, preneural plate, and wider vertebral scales, all which are distinct from the sympatric *S*. *lens* (Fig. 2).

Comparative anatomical studies were conducted through first hand examination of sinemydid species: *Dracochelys bicuspis Gaffney & Ye, 1992* (IVPP V4075 holotype, IVPP V12091; Brinkman, 2001); *Kirgizemys* (=*Hangaiemys*) *hoburensis* (Sukhanov & Narmandakh, 1974) (PIN 3334-4, PIN 3334-1, PIN 3334-5, PIN 3334-16, PIN 3334-34, PIN 3334-35, PIN 3334-36, PIN 3334-37); *Judithemys sukhanovi* Parham & Hutchison, 2003 (TMP 87.2.1 holotype and material listed in Parham & Hutchison, 2003); *Liaochelys jianchangensis Zhou, 2010a* (PMOL-AR00140 holotype, PMOL-AR00160); *Manchurochelys manchoukuoensis Endo & Shikama, 1942* (PMOL-AR00008, AR00007, AR00180, PKUP V1070; Zhou, 2010b; Zhou, Rabi & Joyce, 2014; Shao et al., 2017); *Ordosemys leios Brinkman & Peng, 1993a* (IVPP V9534-1 holotype, and material listed in Brinkman & Peng, 1993a); *Ordosemys liaoxiensis* (Ji, 1995) (IVPP V11554, SDUST-V1020, and material listed in Tong, Ji & Ji, 2004); *Ordosemys* sp. (IVPP V12092, Brinkman & Wu, 1999); *Ordosemys brinkmania Danilov & Parham, 2007* (IVPP V4074.4–holotype and material listed in Danilov & Parham, 2007); *Sinemys gamera Brinkman & Peng, 1993b* (IVPP V9532-1 holotype, IVPP V9532-11 and material listed in Brinkman & Peng, 1993b); *Sinemys brevispinus Tong & Brinkman, 2013* (IVPP V9538-1 holotype); *Sinemys lens Wiman, 1930* (IVPP V8755, IVPP V9533-1, IVPG-T001-5); *Macrobaena mongolica Tatarinov, 1959* (PIN 533-4, holotype); *Xiaochelys ningchengensis Zhou & Rabi, 2015* (PMOL-AR00210AB holotype); *Jeholochelys lingyuanensis Shao et al., 2018* (PMOL-AR00190, AR00211—holotype, AR00213, AR00214, AR00217, AR00218, AR00222). The following species were compared through the literature: *Changmachelys bohlini Brinkman et al., 2013*, *Wuguia hutubeiensis Matzke et al., 2004*; *Wuguia efremovi* (Khosatzky, 1996) ((Danilov & Sukhanov, 2006)); *Asiachelys perforata* Sukhanov & Narmandakh, 2006 (*Ordosemys perforata* sensu Danilov & Parham, 2007); *Manchurochelys donghai* (Ma, 1986) (*Ordosemys donghai* sensu Brinkman, Li & Ye, 2008). See also Sukhanov (2000), Rabi, Joyce & Wings (2010), and Danilov, Syromyatnikova & Sukhanov (2017) for reviews on Mesozoic turtles from Asia.

## Systematic Paleontology

**Table:** 

**Figure 4:**
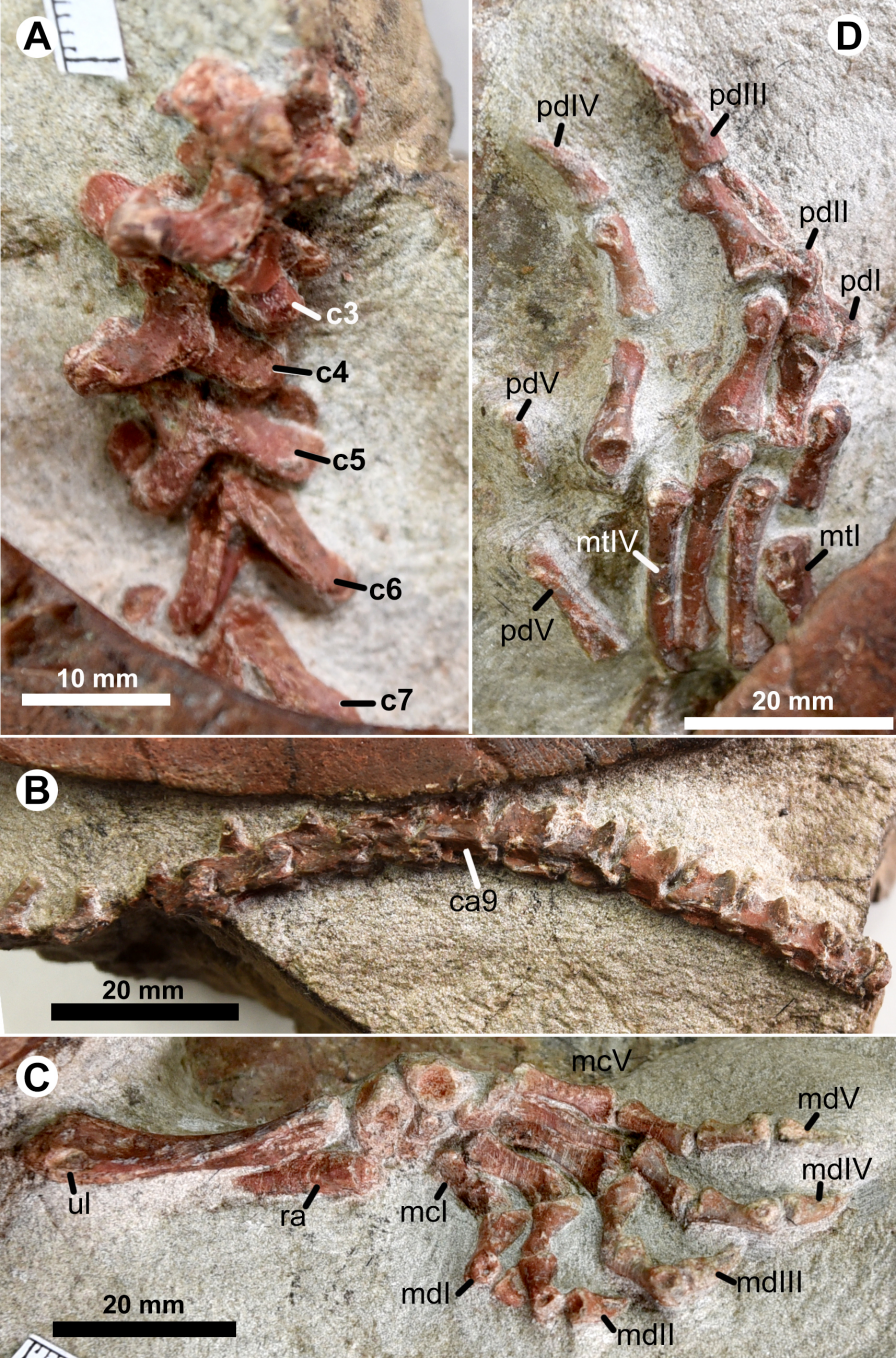
Postcranial skeleton of * Ordosemys leios* (the adult IVPG-T001-1) from the Early Cretaceous Mengyin Formation of Ningjiagou, Xintai, western Shandong, China. Cervical series (A; in anterodorsal view), caudal series (B; in lateral view), left forelimb (C; in dorsal view), left hindlimb (D; in dorsal view). Abbreviations: c3–c7, cervicals 2–7; ca9, caudal 9 in preservation; mcI–mcV, metacarpals I–V; mdI–mdII, manual digits I–II; mtI–mtIV, metatarsals I–IV; pdI–pdV, pedal digits I–V; ra, radius; ul, ulna.

**Figure 5:**
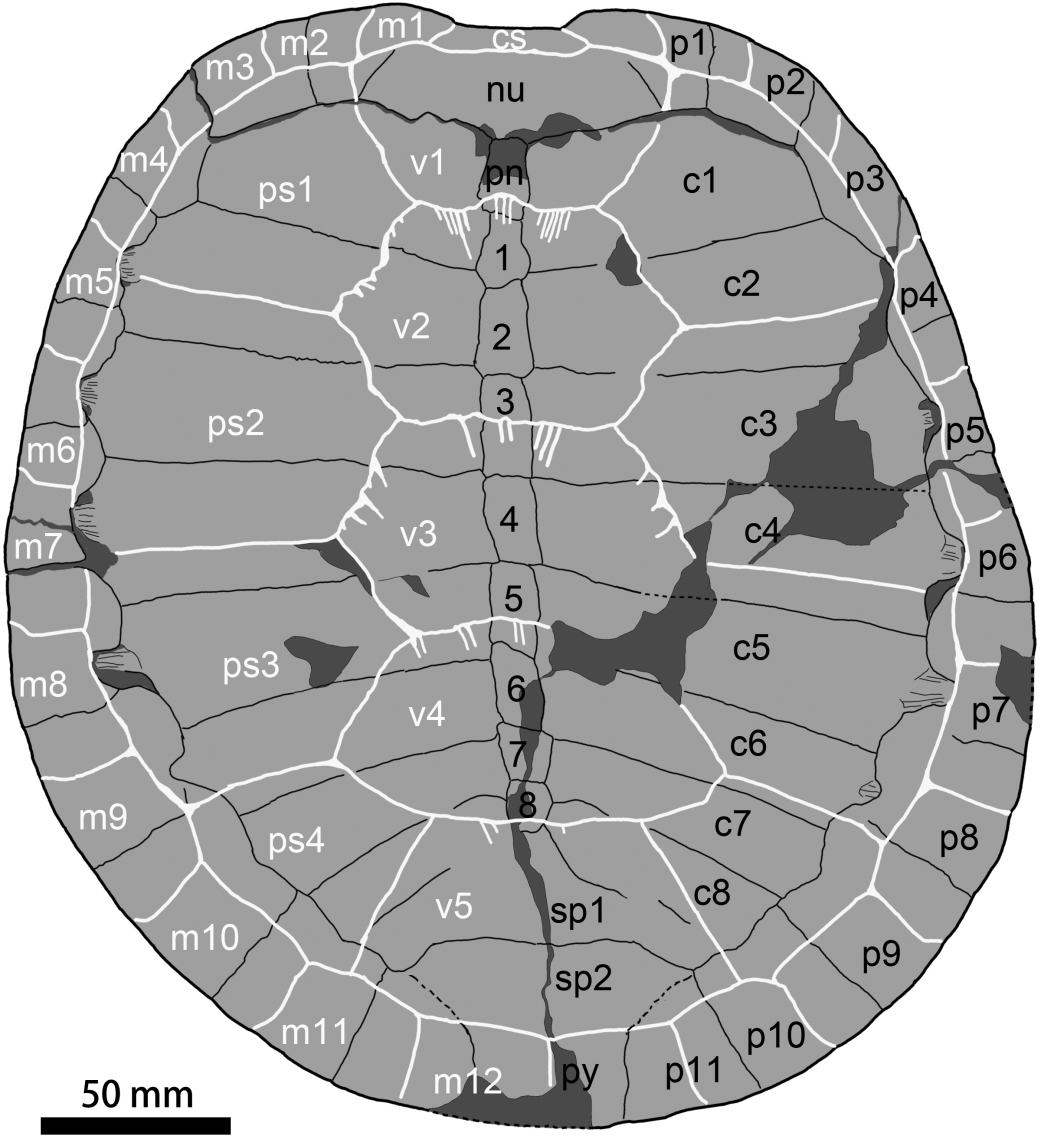
Carapace of * Ordosemys leios* (the adult IVPG-T001-1; in dorsal view) from the Early Cretaceous Mengyin Formation of Ningjiagou, Xintai, western Shandong, China. Abbreviations: 1–8, neural plates 1–8; c1–c8, costal plates 1–8; cs, cervical scale; m1–m12, marginal scales 1–12; nu, nuchal; p1–p11, peripheral plates 1–11; pn, preneural plate; ps1–ps4, pleural scales 1–4; py, pygal; sp1–sp2, suprapygals 1–2; v1–v5, vertebral scales 5.

Testudinata Klein, 1760
Testudines Batsch, 1788
Pan-Cryptodira Joyce, Parham & Gauthier, 2004
Sinemydidae sensu Rabi et al., 2014
*Ordosemys leios* Brinkman & Peng, 1993a
(Figs. 2–5)

**Diagnosis** (revised from Brinkman & Peng, 1993a; Li & Tong, 2017): the combination of a circular carapace, preneural plate, wide vertebrals, central and lateral plastral fenestrae, smooth carapace, deep nuchal emargination, and a median fenestra between the hypoplastron and xiphiplastron. Differs from *O. liaoxiensis* by elongate crista supraoccipitalis, elongated costal 1, possibly a broad contact of the first peripheral and costal, expanded posterior peripherals, and shorter manus; and from *O. brinkmania* by two suprapygals, and possibly a median fenestra between the hypoplastron and xiphiplastron (whether this fenestra closes during ontogeny remains to be tested).

### Referred specimens

IVPG-T001-1, a nearly complete adult skeleton exposed in dorsal view, but missing the cranium and the right limbs. IVPG-T001-2, a nearly complete juvenile skeleton, partially obscured by IVPG-T001-1, and only exposed with the skull, the cervical series and the carapace in dorsal view. IVPG-T001-3, a partial juvenile skeleton, including most part of the shell, cervical series, right forelimb. IVPG-T001-4, a juvenile specimen exposed with most part of the carapace.

### Locality

The slab with the fossil turtles and fishes (*Lycoptera* and *Sinamia*) was collected 500 m North of Ningjiagou village, Xintai City, Shandong Province, Mengyin Formation in November 2012 (35°49′13″N; 117°49′30″E). This locality yielded the earliest discoveries of Mesozoic vertebrate fossils from China. First reported by the Swedish Vertebrate Paleontologist Carl Wiman in 1929, the valuable reptile fossils collected from the fluvial sandstone deposits, include the sauropod *Euhelopus zdanskyi*, the type species of the sinemydid turtles, *Sinemys lens*, the enigmatic pan-cryptodire *Sinochelys applanata* (Wiman, 1929; Wiman, 1930), a stegosaur, pterosaur remains, as well as amiiform and osteoglossomorph fishes (Borinder, Poropat & Kear, 2016). There is growing consensus that the Mengyin Formation belongs to the Lower Cretaceous (Wilson & Upchurch, 2009). Bivalve stratigraphy suggested Berriasian-Barremian (Ma, 1994) whereas tetrapod faunas have been interpreted to indicate Barremian-Aptian age (Wu, Brinkman & Lu, 1994; Dong, 1992; Averianov & Skutschas, 2000; Barrett & Wang, 2007). More recently, detrital zircon and U-Pb zircon dating yielded an age between 145–136 Ma which corresponds to the Berriasian-Valanginian interval (basal Cretaceous, Xu & Li, 2015).

## Comparative Description

### Skull

The skull is exposed in dorsal and lateral views in the juvenile specimen IVPG-T001-2 (Fig. 3). It is three-dimensionally preserved without any distinct deformation. The lower jaw is articulated to the skull and is only exposed in lateral view. The skull has a maximum length of 34.4 mm from the rostral tip to the distal end of the supraoccipital crest, a maximum width of 24.5 mm across the postorbital region, and a maximum depth of 11 mm. The skull has a subtriangular dorsal profile with a longer than high orbit. The interorbital roof is narrower than in *O*. *brinkmania*.

The cranial roof is sculptured by cranial scale sulci. Two pairs of F scales (scalation nomenclature follows Sterli & De la Fuente (2013) and Rabi et al. (2014)) are present on the interorbital roof. The anterior paired F scales meet along the midline. The posterior paired F scales are the largest scales and are completely separated by a single diamond-shaped scale Y. Another median scale, X, is positioned more posteriorly and excluded from contacting scale Y by the small paired G scales. Scale X is slender, has deep sulci, and is followed by scale A posteriorly. Along scale X, the paired D scales extend sagittally and contact the scales G and F anteriorly. More laterally, the sulci are poorly visible in the postorbital region. The scalation pattern differs from the proposed morphology of the xinjiangchelyid *Annemys levensis* (Rabi et al., 2014) in the absence of scales Z, a single pair of scales F anterior to scale Y instead of two pairs, and in the markedly smaller scales G.

The temporal emargination is moderately developed and the processus trochlearis oticum is not exposed in dorsal view. The supraoccipital crest is slightly beyond the posterior end of the squamosal, as in *O*. *brinkmania*, but longer than that of *Ordosemys liaoxiensis*. In lateral view, the cheek emargination is as developed as in the skull of *Ordosemys* sp. described in Brinkman & Wu (1999).

The nasals are small and plate-like at the rostral end of the cranial roof. As in other species of *Ordosemys*, the nasals exclude the prefrontals and frontals from the margin of the external naris.

The sub-rectangular prefrontals are fully separated from each other by the frontals and are much wider than the frontal in the interorbital roof, different from *Ordosemys* sp., in which the prefrontal is relatively narrow, and comparable to the frontal in width (Brinkman & Wu, 1999). At the anterodorsal corner of the orbit, the prefrontal bears a short ventral process along the maxilla.

The frontals are positioned among the nasals and prefrontals anteriorly, the postorbitals laterally and the parietals posteriorly. Anteriorly, the frontal extends a rostral process. The process is slender and completely separates the prefrontals. Behind the prefrontal, the frontal has a limited contribution to the orbital margin.

Laterally, the parietal bears a lateral process to contact the squamosal and together they form the anterior margin of the upper temporal emargination (to the exclusion of the postorbital). A similar condition is present in *O*. *liaoxiensis* and possibly in *Ordosemys* sp. where the shape of the posterolateral process of the parietal suggest that it was in contact with the squamosal. However, this region is poorly preserved in *O. brinkmania*.

The premaxillae form the ventral rim of the naris together with the maxillae. The ventral margin of the maxilla is relatively smooth, lacking the tooth-like process in *Dracochelys bicuspis*. Dorsally, the maxilla forms the anterior and ventral margins of the orbit and bears a dorsal process to contact the nasal and prefrontal. The contact of the jugal with the quadratojugal posteriorly is uncertain, due to the damaged cheek emargination (Fig. 3C). The quadratojugal-jugal connection is present in *O*. *liaoxiensis* and *Ordosemys* sp. (e.g., Brinkman & Wu, 1999; Tong, Ji & Ji, 2004).

The quadratojugal is triradiate, anteriorly forms the cheek emargination, posteriorly embraces the quadrate and builds a possible contact with the squamosal (Fig. 3C). The possible quadratojugal-squamosal contact separates the postorbital from the quadrate, unlike in *O*. *liaoxiensis* and *Ordosemys* sp.

Through the upper temporal emargination, the cranial elements (e.g., prootic, opisthotic, and quadrate) are poorly discernible, except for the foramen stapedio-temporale (Figs. 3A, 3B).

### Axial skeleton

The cervical series is exposed in articulation in IVPG-T001-1 and IVPG-T001-2. In the adult IVPG-T001-1, cervicals 3 to 7 are preserved (Fig. 4A); while the anterior four cervicals (the atlas, the axis, cervicals 3 and 4) are exposed in IVPG-T001-2 (Figs. 3A, 3B). In IVPG-T001-2, paired atlas neural arches are observable in dorsal view. They are partially obscured by the overlapping supraoccipital crest. The arch is plate-like and has a short lateral spine. The axis is longer than the succeeding cervicals 3 and 4. It has a blade-like neural spine, and laterally-expanded postzygapophyses. The neural spine is well developed along the midline of the axis, and longer than that of the succeeding cervicals 3 and 4. The postzygapophyses extend more laterally than posteriorly, forming a concave posterior margin of the neural arch. Dorsally, the postzygapophysis bears a low ridge. The ridge extends anteromedially and meets the counterpart at the posterior end of the neural spine. The conjoint ridges are widely angled and roughly parallel to the posterior margin of the neural arch. They are linked by a tiny ridge developed along the midline. The transverse processes are anteriorly positioned, as in the succeeding cervicals. The third and fourth cervicals are comparable in size and morphology. Between the prezygapophyses, the anterior notch of the neural arch is developed and comparable to the posterior one. In the adult IVPG-T001-1, the third cervical appears to have an anterior condyle. In cervicals 5 to 7, the neural spine is further shortened. The postzygapophyses are well developed, and extend more posteriorly than laterally. Medially, they are deeply divergent, forming a V-shaped posterior margin of the neural arch. Dorsally, the conjoint ridges are more developed and more sharply angled than the posterior margin. Cervical ribs are not present in any of the specimens.

The caudal series is partially preserved with 17 caudal vertebrae in articulation in the adult IVPG-T001-1 (Fig. 4B). The posterior caudals appear to be opisthocoelous. The chevra are developed and positioned close to the posteroventral corner of the centrum along the tail.

### Appendicular skeleton

In the adult IVPG-T001-1, the right forelimb is exposed except for the humerus (Figs. 2 and 4C). The ulna is about 34.5 mm long. The intermedium and the medial central carpal of the manus are subequal in width, but the intermedium is more robust and have a roughly triangular profile in dorsal view. In contrast, the ulnare is circular and reduced in size. Two distal carpals are exposed and positioned close to the metacarpals IV and V.

The manus is moderately elongate: metacarpal III (15 mm) and manual digit III (29.5 mm) have a total length of 44.5 mm, about 129% of the length of the ulna, less than that of *O. liaoxiensis* (Manus/ulna = 167%; estimated from Tong, Ji & Ji, 2004: fig.9). In contrast, the manus is longer, nearly twice of the ulnar length, in other sinemydids where the forelimb is known (e.g., *Xiaochelys ningchengensis*, *Changmachelys bohlini* and *Jeholochelys lingyuanensis*; Brinkman et al., 2013; Zhou & Rabi, 2015; Shao et al., 2018). The metacarpus is exposed in dorsal view and its elements are loosely arranged and slightly displaced. Metacarpal I is shorter and more robust than other metacarpals. Metacarpals II–V are slender and elongated. Of these, metacarpals III and IV share a maximum length of 15 mm. Metacarpals I to III are flattened dorsoventrally and they contrast the rod-like metacarpals IV and V in having a sharp medial side along the shaft. Distally, metacarpals I–III are expanded. On the lateral face of the distal condyles, a small fossa is developed which is absent in metacarpal V and possibly in metacarpal IV. The five digits are clawed with a phalangeal formula of 2-3-3-3-3. Digits III and IV are elongate and subequal in length (29.5 mm). The proximal phalanges are more robust than the middle phalanges. The proximal phalanx bears a cotyle for the metacarpal. Ventral to the cotyle, a well-developed process extends proximally to form the maximum depth of the phalanx. This ventral process is poorly developed in the middle phalanges. The middle phalanges are slightly shorter than the proximal phalanges. The unguals of digits III and IV are slightly curved and longer than the associated middle phalanges.

The hind limb is only exposed with the left pes (Figs. 2 and 4D). The pes is partially hidden by the carapace, and has a limited exposure in dorsal view. The pes is larger and more robust than the manus. Metatarsal I is the shortest and widest. It is relatively flat dorsoventrally. Metatarsals II–IV are elongate and rod-like. Of these, metatarsal III is the longest with a length of 21 mm. The ansulate bone is hooked, and isolated from the other metatarsals. The phalangeal formula of the pes is 2-3-3-3-?. The digit III is the longest with a length of 38.2 mm. Digit V only preserves two phalanges and its phalangeal number is uncertain. The pedal phalanges are comparable with the manual phalanges in morphology, but longer than the latter. The claws in digits I–IV are more robust than the manual claws.

### Carapace

The carapace is subcircular (Figs. 2 and 5), as that of *Ordosemys* spp., *Changmachelys bohlini*, and *Xiaochelys ningchengensis*. In the adult IVPG-T001-1, the carapace has a maximum length of 260 mm as preserved (estimated total length in life is 263 mm), and a maximum width of about 244 mm at the level of the fourth costal plates. A longitudinal midline depression is developed along the neural region as in other sinemydids (e.g., *Sinemys* spp.; *Manchurochelys manchoukuoensis*; *Liaochelys jianchangensis*; *Jeholochelys lingyuanensis* and *Judithemys sukhanovi*). The carapacial surface is generally smooth. The anterior third of the vertebrals are ornamented with radially arranged plications in both the adult and juvenile specimens. Such plications are absent in the other known species of *Ordosemys,* except for a weakly developed condition on the neural plate of IVPP V4074.14 in *O*. *brinkmania*.

The nuchal plate is wide and forms a distinct emargination which is more pronounced in the adult IVPG-T001-1 than in the juveniles. The nuchal contacts the first peripherals laterally, the first costals posterolaterally, and the preneural posteriorly. In contrast, a point contact of the nuchal and second peripherals is present in *O*. *liaoxiensis* and *D*. *bicuspis* whereas in *O. leios* (Ordos Basin) it is variable (point-like in IVPP V9534-1 and broad contact in V9534-3). Dorsally, the nuchal is covered by the scales of cervical and the first marginal anteriorly and the first vertebral posteriorly. In the juveniles, nuchal fontanelles are present between the first three peripherals and the first costals.

A preneural plate is present, which is unique to *Ordosemys* spp. among sinemydids. In the adult IVPG-T001-1, the preneural is partially broken on its surface, but its profile can be traced. It is sub-rectangular, longer than wide, and comparable to the first neural in size. In the juvenile IVPG-T001-2, however, the preneural is wider than long, and smaller than the first neural. Posteriorly, the preneural is crossed by the intervertebral sulcus of the vertebrals 1 and 2. In IVPG-T001-2, the sulcus is confluent with the preneural-neural suture.

The neural series consist of eight elements. The first neural is roughly rectangular with irregular sides. Neural 2 is sub-pentagonal with very short posterolateral sides. Neural 3 is the largest element. It is rectangular and crossed by the sulcus of vertebrals 2 and 3. Neurals 4 to 6 are sub-rectangular, and decrease in size along the series. Neurals 7 and 8 are sub-pentagonal with short anterolateral sides. In the juvenile specimen IVPG-T001-2, the neurals are uniformly rectangular in shape. As in the adult IVPG-T001-1, the neural series size variation is 1<2<3>4>5>6>7>8.

Behind the neural series, the bony sutures are difficult to identify around the surprapygal region. Two suprapygals are possibly present. The pygal is distally incomplete.

Eight pairs of costal plates are present. The costo-peripheral fenestrae are fully closed in the adult IVPG-T001-1, while a half-closed condition is present in the other three individuals (IVPG-T001-2, IVPG-T001-3, and IVPG-T001-4; Fig. 2) as a juvenile feature that is also observed in other sinemydids (e.g., Tong, Ji & Ji, 2004; Shao et al., 2017). The first costal plate is anterolaterally directed. Its proximal margin is longer than that of the more posterior costals. Costal 2 is wider than the first costal, and slightly expanded distally. Its distal end has a slight exposure within peripheral 4 in dorsal view. A similar condition is present in costals 3 to 6. This feature is widely distributed in the Early Cretaceous sinemydids (Sukhanov, 2000; Brinkman, 2001; Tong, Ji & Ji, 2004; Zhou, 2010a; Zhou, 2010b; Tong & Brinkman, 2013; Zhou & Rabi, 2015). Costal 3 is the largest costal plate with parallel anterior and posterior sides. Distally, costal 3 is wedged between peripherals 5–6, its terminal is partially exposed within peripheral 5. Costal 4 is comparable with costal 3 in size. The succeeding costals are gradually reduced in size and directed obliquely.

Eleven pairs of peripheral plates form the carapace with the nuchal and the pygal. As in *O*. *leios* and *O*. *liaoxiensis*, the gutter (e.g., Rabi et al., 2014) is absent along the anterior peripherals. Peripheral 1 is small and subtriangular, and has a broad contact with the first costal. The succeeding peripherals 2 and 3 are enlarged. Peripheral 2 have a rectangular outline, while peripheral 3 is sub-pentagonal with an angular medial side. The succeeding peripherals 4 to 6 become more slender, and slightly expanded medially. In contrast, the posterior peripherals 7 to 11 are distinctly enlarged and well expanded beyond the pleural-marginal sulci, different from the reduced peripherals of *O. liaoxiensis*. In peripherals 4 to 8, the medial side bears a distinct notch that is occupied by the distal end of associated costal. The notch gradually enlarges from peripheral 4 to peripheral 7, and then strongly reduces in peripheral 8. The notch is absent in the last peripherals 9 to 11, so that the distal ends of costals 7 and 8 are unexposed in dorsal view.

### Carapacial scales

The carapacial scale sulci are deeply impressed (Figs. 2 and 5). The cervical scale is slender and limited to the nuchal emargination. The vertebrals are much wider than long, similarly to other species of *Ordosemys*. Their sides are somewhat sinuous and anteriorly ornamented with plications. In contrast, the vertebrals are smooth with nearly straight sides in *O*. *leios*, *O*. *liaoxiensis*, and *O*. *brinkmania*. Inter-vertebral sulci are proportionally shorter relative to that of other species of *Ordosemys*.

Vertebral 1 is trapezoid with a longer anterior side. It has a short contact with marginals 2. Vertebral 1 is as wide as the nuchal, and extends on the nuchal, peripheral 1, costal 1 and the preneural. Vertebrals 2 to 4 are hexagonal, and slightly wider than vertebrals 1 and 5. Vertebrals 2 and 3 have comparable sizes to each other, and vertebral 4 is slightly smaller. Vertebral 2 has notably sinuous lateral sides. The vertebral 1–2 sulcus has a small anterior midline projection across the preneural (absent in the juvenile IVPG-T001-2 and T001-3). A similar condition is also present in *O*. *brinkmania* and a referred specimen of *O. leios* (IVPP-V-9534-3). Along the sulcus, several plications are developed and posteromedially directed. Similar sulcus plications are also present in other inter-vertebral sulci. The vertebral 2–3 sulcus is slightly wider than the vertebral 1–2 sulcus, and crosses the middle part of neural 3. The vertebral 3–4 sulcus crosses the posterior portion of neural 5. The posterior side of vertebral 4 is reduced, and its posterolateral sides are shorter than the anterolateral sides. Vertebral 5 is trapezoid and has a longer, curved posterior side. Vertebral 5 is larger than the suprapygals and extends onto peripherals 10 and 11 laterally.

Pleurals 1 to 3 are wider than long. Pleurals 2 and 3 are subequal in width. Their width is comparable to that of vertebrals. Pleural 4 is reduced in size and has a similar width and length.

The marginals increase in size posteriorly. They are restricted to the peripherals, except for the first marginals that extend onto the nuchal. Marginal 2 has a short contact with vertebral 1, as in *O*. *leios* (IVPP V93534-3), unlike the point contact in *O*. *leios* (IVPP V93534-1, the holotype), *O*. *liaoxiensis*, and *Judithemys sukhanovi*. The pleuro-marginal sulci of marginals 4 to 6 coincide with the costo-peripheral suture; whereas the pleuro-marginal sulci of marginals 7 to 11 are limited to the associated peripherals. Marginals 12 meet each other along the midline and cover the pygal at the distal end of the carapace. The vertebral 5—marginal 12 sulcus is coinciding with the pygal—suprapygal suture.

## Discussion

### Taxonomy

Among sinemydids, the new specimens from the Mengyin Formation share the typical characters of *Ordosemys*, including the circular carapace, the preneural plate, and the wide vertebrals (Brinkman, Li & Ye, 2008). Five species has been referred to *Ordosemys* including *O*. *leios*, *O*. *liaoxiensis*, *O*. *brinkmania*, *O*. *perforata*, and *O*. *donghai*. The combined presence of an elongate crista supraoccipitalis, an elongated costal 1, expanded posterior peripherals, and a shorter manus separates the Mengyin taxon from *O. liaoxiensis*. The long contact between peripheral and costal 1 and the likely correlated exclusion of the pleural from the nuchal in the Mengyin fossil further differentiates it from *O. liaoxiensis* but these characters may be intraspecifically variable in a larger sample (e.g., cf. IVPP-V 9534-1 and 9534-3 of *O. leios*). The Mengyin taxon is also different from *O. brinkmania* in having two suprapygals. Two suprapygals are common in *O. liaoxiensis* (Ji, 1995; Li & Liu, 1999; Tong, Ji & Ji, 2004) and *O. leios* (Brinkman & Peng, 1993a), but three suprapygals are only known *O. brinkmania* (Danilov & Parham, 2007). Furthermore, *O. brinkmania* may differ from *O. leios* by the absence of a median fenestra between the hypoplastron and xiphiplastron (Danilov & Parham, 2007) although it remains unclear if the fenestra closes during ontogeny. Unfortunately, the plastral structure is unexposed in the Mengyin fossils of *O. leios* therefore hindering a further comparison with *O. brinkmania*. The only differences we can observe between the carapace of the Mengyin form and *O. leios* from the type horizon are the development of vertebral plications, the shorter intervertebral sulci, and the subequal-sized preneural-neural in the former. However, both localities yielded few specimens only and until intraspecific variability is better explored we find that the available Mengyin material does not warrant defining a new taxon. We therefore tentatively refer these specimens to *Ordosemys leios.* Further material from the type horizon of *O. leios*, as well as complete preparation of the material from Mengyin Formation, would likely yield insights into taxonomy. The isolated skull described by Brinkman & Wu (1999; IVPP V12092) from the type horizon of *O*. *leios* (Luohandong Formation) differs from the juvenile Mengyin specimen in the absence of scale sulci. We are unsure whether cranial scale sulci can disappear with ontogeny and we refrain from diagnosing a new species based on this character. Although Brinkman & Wu (1999) left the species attribution of this isolated skull unresolved (referred to *Ordosemys* sp.), the reported morphological differences from the poorly known skull of the holotype of *O. leios* are either nuances or hard to reproduce. Likewise, the shell material of *O*. *leios* from the type locality area is not showing variation that could not be assigned to intraspecific or preservational difference. Taken all this into account, together with the great similarity of the isolated skull IVPP V12092 to that herein described of *Ordosemys leios* from the Mengyin Formation, we find the presence of a single species of *Ordosemys* at the Laolonghuoze area of the Luohandong Formation the most parsimonious based on current evidence.

Two species, *O*. *perforata* (=*Asiachelys perforata* sensu Sukhanov & Narmandakh, 2006; Danilov & Parham, 2007) and *O*. *donghai* (=*Manchurochelys donghai* sensu Ma, 1986; Brinkman, Li & Ye, 2008; Li & Tong, 2017) have been questioned in their affinities to *Ordosemys* in lacking a preneural plate (e.g., Tong, Ji & Ji, 2004; Danilov & Parham, 2007; Li & Tong, 2017). *Ordosemys perforata* from the Early Cretaceous Khulsangol Formation of Khuren Dukh, Mongolia, known from an incomplete plastron, is similar to *Ordosemys* spp. in plastral proportions, as well as having central, lateral and hypo-xiphiplastral fenestrae (Danilov & Parham, 2007) but these characters are present in other sinemydids including *Liaochelys jianchangensis* and *Changmachelys bohlini* (Zhou, 2010a; Brinkman et al., 2013) and are subject to ontogenetic variation (e.g., Shao et al., 2018). The affinity of *O*. *perforata* with *Ordosemys* (sensu Danilov & Parham, 2007) therefore remains to be confirmed. *O*. *donghai* (Ma, 1986; *Manchurochelys donghai*) is based on an incomplete carapace from the Early Cretaceous Chengzihe Formation of Jixi, Helongjiang, China. It was referred to *Ordosemys* by Brinkman, Li & Ye (2008) though no justification was provided (see also Li & Tong, 2017). Based on the original illustration, *O*. *donghai* is different from *Ordosemys* spp. in the absence of a preneural and the only slightly wider than long vertebral scales which questions the proposed affinity of this species. It does share, however, the presence of vertebral plications with the Mengyin form but this character is known in many other Mesozoic pan-cryptodires from Asia (e.g., Rabi, Joyce & Wings, 2010).

### Paleoecological implications

**Figure 6:**
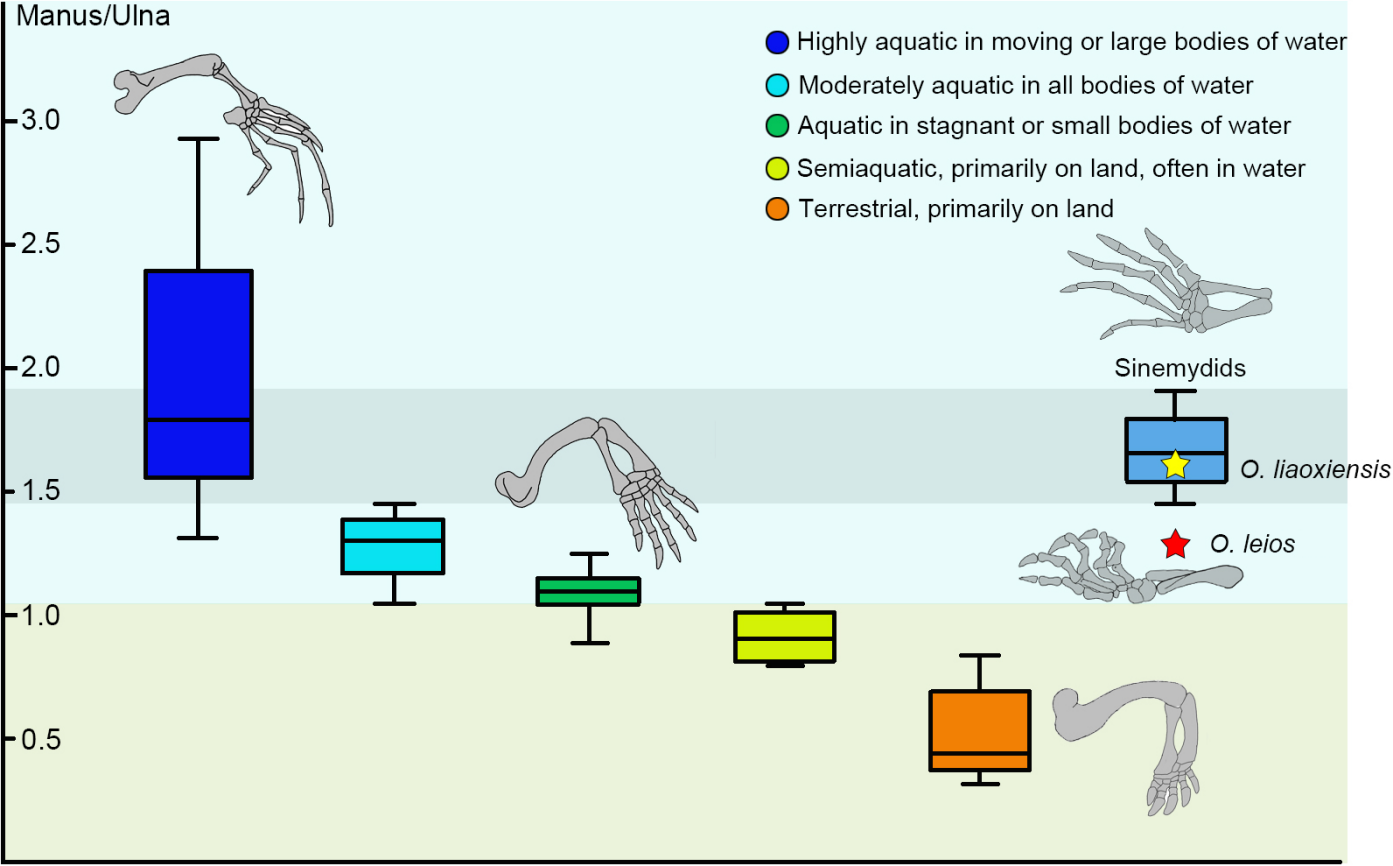
Box plots for the manus-ulna proportion of the sinemydids and extant turtles. The box plots performed by PAST 2.12 (Hammer, Harper & Ryan, 2001), and the extant data from Joyce & Gauthier (2004).

The relationship between ecology and relative manus length is well established in extant turtles and can be used to infer ecology in fossil taxa (Joyce & Gauthier, 2004). In sinemydids known from articulated skeletons (e.g., *Changmachelys bohlini*, *Xiaochelys ningchengensis*, *Ordosemys liaoxiensis*, *Jeholochelys lingyuanensis*), the manus is elongated and corresponds to 165–179% of the length of the ulna which is comparable to that of the highly aquatic soft-shell turtles (Shao et al., 2018; Fig. 6).

In *Ordosemys* spp., the forelimbs have been so far only known in specimens coming from the laminated deposits of the Yixian Formation (*O*. *liaoxiensis*). The proportion of the manus relative to the ulna is estimated to reach 167% in *O*. *liaoxiensis*, based on the illustration of Tong, Ji & Ji (2004: fig.9) which is further confirmed by other specimens of *O*. *liaoxiensis* (GM V3000-1, Tong, Ji & Ji, 2004; SDUST-V1020, Shao et al., 2018). This proportion contrasts with the shorter manus of *O*. *leios* from the Mengyin Formation where it is about 129% the length of the ulna and thus corresponds to the “moderately aquatic” category of Joyce & Gauthier (2004; Fig. 6). Species belonging to this category inhabit a wide range of water bodies and include most extant freshwater turtles. Given the significant bias towards preservation of articulated turtles in laminated lacustrine deposits in the Lower Cretaceous of China (Tong, Ji & Ji, 2004; Brinkman et al., 2013; Zhou & Rabi, 2015; Shao et al., 2018), the articulated limbs of *Ordosemys leios* coming from fluvial sandstone deposits of the Mengyin Formation provides important data for potentially testing ecomorphological-environmental patterns among sinemydids in the future.

### Implications for the age of the Luohandong Formation

**Figure 7:**
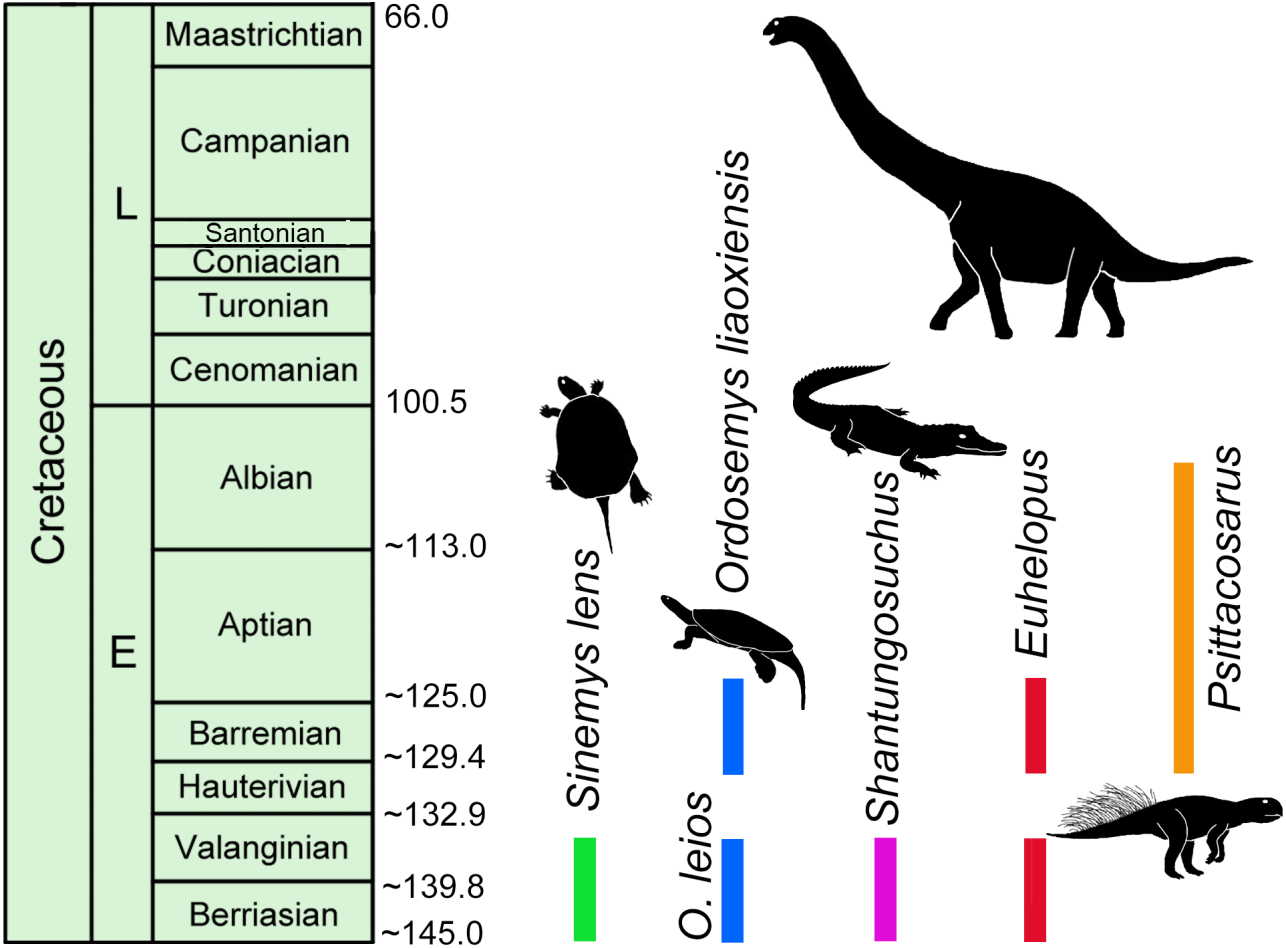
Temporal distribution of *Sinemys lens*, *Ordosemys* spp., *Shantungosuchus*, *Euhelopus* and *Psittacosaurus* based on radiometrically dated fossil-bearing deposits. The 145–136 Ma zircon age of the Mengyin Formation is after *Xu & Li (2015)*. The sanidine ages of the basalt and tuff layers of the Yixian Formation of the Jehol Biota yielded 130.6–122.9 Ma (Chang et al., 2009; Chang et al., 2017). *Euhelopus* spp. the temporal distribution of *Psittacosaurus* is so far restricted to an age interval between 130.6 Ma (at the bottom of Yixian Formation, (Chang et al., 2009; Chang et al., 2017) and 105 Ma (at the top of Qingshan Group (Ar age of Zhang et al., 2008; Young, 1958; Lucas, 2006). The younger record of *Euhelopus* is after Barrett & Wang (2007).

Absolute dating studies on Lower Cretaceous units in China are few (e.g., Yixian Formation and Jiufotang Formation, 130.6–120 Ma; He et al., 2004; Chang et al., 2009; Chang et al., 2017) and thus establishing relative ages are often left to rely on preliminary vertebrate biostratigraphy (e.g., Lucas, 2006; Hou et al., 2017). Sinemydid turtles are among the most common and best preserved elements of these biotas and, though sampling is limited, the only formations where *Sinemys* and *Ordosemys* are known to co-occur are the Mengyin Formation of Shandong province and the Luohandong Formation of the Ordos Basin in Inner Mongolia. *Ordosemys leios* represents the third turtle taxon from the Mengyin Formation in addition to *Sinemys lens* and *Sinochelys applanata*. The Mengyin turtle fauna is thus most similar to that of the Luohandong Formation of the Ordos Basin, the type and only horizon of *Ordosemys leios* and *Sinemys gamera* (*Sinemys brevispinus* has been recently reinterpreted to originate from the overlying Jingchuan Formation; Ji & Chen, 2018). *Euhelopus*-like sauropod teeth (Hou et al., 2017) and the crocodyliform *Shantungosuchus* from the Luohandong Formation (Wu, Brinkman & Lu, 1994) further suggest a faunal composition that is otherwise only known from the Mengyin Formation (Borinder, Poropat & Kear, 2016; Fig. 7). On the other hand, Hou et al. (2017) erroneously cited Young (1958) that *Psittacosaurus* is also common to both formations with *P. sinensis* reported from the Mengyin Formation; in fact, this information is not present in Young’ paper (1958) (Fig. 7).

No absolute dates have been published from the the Luohandong Formation and previous biostratigraphic studies disagree: it has been considered Valanginian—early Hauterivian (Chen et al., 2006), Barremian (Averianov & Skutschas, 2000), or Aptian (Wan et al., 2013; Li, 2017) in age. The Mengyin Formation has been recently dated to 145–136 Ma, corresponding to the Berriasian-Valanginian using detrital zircons (Xu & Li, 2015; Fig. 7)*.* If this date is correct, the *Sinemys*-*Ordosemys* community and the other mentioned faunal similarities are most consistent with a Valanginian—early Hauterivian age for the Luohandong Formation (as proposed by Chen et al., 2006, p. 28 based on dinoflagellate, floral, and bivalve correlation). On the other hand, a Valanginian—early Hauterivian age is inconsistent with the presence of the ceratopsid dinosaur, *Psittacosaurus*, in the Luohandong Formation (e.g., Brinkman & Peng, 1993a; Lucas, 2006; Tong & Brinkman, 2013; Hou et al., 2017) as this taxon is used for defining a biochron between two radiometrically dated horizons: the Yixian / Jiufotang formations (130.6–120 Ma; He et al., 2004; Chang et al., 2009) and the Qingshan Group (120–105 Ma; Zhang et al., 2008; Lucas, 2006). This interval corresponds to the late Hauterivian-Albian (Fig. 7). Three possible scenarios can explain this controversy: (1) either the Mengyin Formation is younger than what the radiometric age result of Xu & Li (2015) suggests; or the fossil record of the *Ordosemys-Sinemys* community (2) or *Psittacosaurus* (3) is biased. Since the Mengyin Formation yielded the only radiometrically dated vertebrate fauna older than the *Psittacosaurus-* biochron, and since low collecting intensity precludes ruling out the presence of *Psittacosaurus* within this horizon, it seems impossible to test between these hypotheses at the moment. Further absolute dates and fossils from the Lower Cretaceous of China will be vital for resolving these questions.

## Institutional Abbreviations

IVPG: Institute of Vertebrate Paleontology, Gansu Agricultural University, Lanzhou, China
IVPP: Institute of Vertebrate Paleontology and Paleoanthropology, Beijing, China
PIN: Paleontological Institute, Russian Academy of Sciences, Moscow, Russia
PMOL: Paleontological Museum of Liaoning, Shenyang Normal University, Shenyang, China
PKUP: Paleontological Collections, School of Earth and Space Sciences, Peking University, Beijing, China
SDUST: College of Earth Science and Engineering, Shandong University of Science and Technology, Qingdao, China
TMP: Royal Tyrrell Museum, Drumheller, Alberta, Canada

## Additional Information and Declarations

### Data Availability

The following information was supplied regarding data availability: The specimens of *Ordosemys* described in this study are stored in Institute of Vertebrate Paleontology and College of Life Science and Technology, Gansu Agricultural University, Lanzhou, China. Accession numbers: IVPG-T001-1, IVPG-T001-2, IVPG-T001-3, IVPG-T001-4.
